# Myriocin Significantly Increases the Mortality of a Non-Mammalian Model Host during *Candida* Pathogenesis

**DOI:** 10.1371/journal.pone.0078905

**Published:** 2013-11-15

**Authors:** Nadja Rodrigues de Melo, Ahmed Abdrahman, Carolyn Greig, Krishnendu Mukherjee, Catherine Thornton, Norman A. Ratcliffe, Andreas Vilcinskas, Tariq M. Butt

**Affiliations:** 1 College of Medicine, Swansea University, Singleton Park, Swansea, United Kingdom; 2 Department of Biosciences, College of Science, Swansea University, Singleton Park, Swansea, United Kingdom; 3 Institut für Phytopathologie und Angewandte Zoologie, Abteilung Angewandte Entomologie, Gieβen, Germany; 4 Department of Biological Sciences, Universidade Federal Fluminense, Rio de Janeiro, Brazil; Instituto de Salud Carlos III, Spain

## Abstract

*Candida albicans* is a major human pathogen whose treatment is challenging due to antifungal drug toxicity, drug resistance and paucity of antifungal agents available. Myrocin (MYR) inhibits sphingosine synthesis, a precursor of sphingolipids, an important cell membrane and signaling molecule component. MYR also has dual immune suppressive and antifungal properties, potentially modulating mammalian immunity and simultaneously reducing fungal infection risk. Wax moth (*Galleria mellonella*) larvae, alternatives to mice, were used to establish if MYR suppressed insect immunity and increased survival of *C. albicans*-infected insects. MYR effects were studied *in vivo* and *in vitro*, and compared alone and combined with those of approved antifungal drugs, fluconazole (FLC) and amphotericin B (AMPH). Insect immune defenses failed to inhibit *C. albicans* with high mortalities. In insects pretreated with the drug followed by *C. albicans* inoculation, MYR+*C. albicans* significantly increased mortality to 93% from 67% with *C. albicans* alone 48 h post-infection whilst AMPH+*C. albicans* and FLC+*C. albicans* only showed 26% and 0% mortalities, respectively. MYR combinations with other antifungal drugs *in vivo* also enhanced larval mortalities, contrasting the synergistic antifungal effect of the MYR+AMPH combination *in vitro*. MYR treatment influenced immunity and stress management gene expression during *C. albicans* pathogenesis, modulating transcripts putatively associated with signal transduction/regulation of cytokines, I-kappaB kinase/NF-kappaB cascade, G-protein coupled receptor and inflammation. In contrast, all stress management gene expression was down-regulated in FLC and AMPH pretreated *C. albicans* -infected insects. Results are discussed with their implications for clinical use of MYR to treat sphingolipid-associated disorders.

## Introduction

Myriocin (2-amino-3,4-dihydroxy-2-(hydroxymethyl)-14-oxoicos-6-enoic acid, MYR) is a metabolite of the insect pathogenic fungus *Isaria sinclairii*. It is a sphingosine analog with immunosuppressive properties, more potent than cyclosporine [1]. MYR also known as ISP-1 and thermozymocidin, has antifungal antibiotic properties [2]. Fingolimod (FTY720) is a novel immune regulatory drug (trade name Gilenya, Novartis) derived from MYR which has been approved for treating multiple sclerosis, a chronic autoimmune disease that affects millions of people worldwide [3]. Besides immune modulation, MYR depletes sphingolipids from cells, by inhibiting the enzyme serine palmitoyltransferase which catalyses the formation of sphingosine, a precursor of sphingolipids [4]. MYR is also an agonist of the sphingosine 1 phosphate receptor [5]. Sphingolipids produce the outer leaflet of the plasma membrane and have a role in protecting the cell surface against harmful environmental factors. Sphingolipid metabolites, such as ceramide and sphingosine-1-phosphate, are important mediators in the signaling cascades involved in apoptosis, proliferation, and stress responses [6]. Since MYR shows promise in treating a number of major diseases additional to multiple sclerosis, including diabetes, cardiovascular disease [7], [8], certain cancers [9], photoreceptor degeneration in retinitis pigmentosa [10] and artherosclerosis [11], it is anticipated that the use of MYR and its derivatives will increase. Therefore, it is imperative that we understand the full potential of the drug including antagonism and synergy with other therapeutics.

*Candida* species are ranked amongst the most common causative agents of invasive fungal infections, with *Candida albicans* being the most frequently isolated species in hospitals worldwide [12]. Treating candidiasis can be costly and can extend a patient's stay in hospital [13]. The incidence of invasive candidiasis is increasing due to the growing number of immune compromised and debilitated patients, the mortality attributable to candidiasis remaining high [14]. It is unclear if the immune suppressive properties of MYR might increase the risk to *Candida* infections or if the antifungal properties provide sufficient protection to reduce the use of other antifungal drugs. Identifying synergistic compounds for combination antifungal therapy offers a promising antifungal strategy [15].

Typically, mammalian model systems are used (e.g. a murine infection model) to evaluate new therapeutics, but these experiments are time-consuming, costly and require full ethical consideration. The larvae of the greater wax moth, *Galleria mellonella*, have emerged as a surrogate alternative model host for human pathogens including fungi [16]. *G. mellonella* is particularly suited to investigating fungal virulence, evaluating the efficacy of antibiotics and studying immune defense responses, including elements of innate immunity shared by both humans and insects [17], [18]. Correlations have been established previously between the pathogenicity of microbes, such as *C. albicans*, *Aspergillus fumigatus*, *Bacillus thuringiensis* and *Pseudomonas aeruginosa*, in *Galleria* and mice [19], [20]. Furthermore, the efficacy of licensed antimicrobial agents has been evaluated in *G. mellonella*, demonstrating remarkable correlation between *in vitro* susceptibility testing results and *in vivo* drug efficacy in both insects and mammals [16].

The aim of this study was to determine if MYR, because of its dual (immune suppression and antifungal) attributes, suppressed the insect immune system and concomitantly prevented candidiasis, or if by weakening the immune system it increased susceptibility to infection. Furthermore, we wanted to establish if MYR combinations with current antifungal agents, amphotericin B (AMPH) and fluconazole (FLC), worked synergistically and increased host survival. In addition, we investigated which elements of the insect immune and stress management system could be used as early warning indicators of stress or infection. The findings of this work could have important clinical implications for prophylaxis of mycoses in immune-compromised patients caused by *C. albicans* and other fungal pathogens, including strains resistant to current antifungal drugs.

## Materials and Methods

### Insect rearing

*G. mellonella* were reared in strict isolation at 28°C, 60% relative humidity, with a 12∶12 h light∶dark cycle. Larvae were maintained on an artificial diet (containing organic wheat bran meal, bees wax, honey, ground rat food, and glycerin) [21].

### Culture and maintenance of *Candida albicans*

The yeast form of *Candida albicans* SC5314 was produced on yeast potato extract agar at 37°C. Individual colonies were harvested at 24 h post-incubation, washed twice in phosphate buffer saline (PBS) pH 7, before determining cell numbers in a hemocytometer.

### Antifungal effect of myrocin on *Candida albicans* growth *in vitro*

To investigate the antifungal effect *in vitro* of MYR on *C. albicans* growth, the antifungal susceptibility tests for MYR, AMPH and FLC were performed in the presence of *C. albicans*. Minimum inhibitory concentrations (MICs) were determined by using the modified broth microdilution method of the Clinical and Laboratory Standards Institute [22]. The antifungal efficacy of MYR, FLC, and AMPH were tested at concentrations ranging from 16 to 0.03 µg/ml for MYR and AMPH and 64-0.01 µg/ml for FLC. The MIC was defined as the lowest concentration of antifungal agent at which growth was inhibited by 80% compared with that of the growth in the control well.

### Drug interactions

The *in vitro* drug interactions were studied by a chequerboard microdilution method [23] which consists of a two dimensional array of serial concentrations of the test compounds. For the combination testing, each drug (MYR, FLC and AMPH) was serially diluted with the corresponding solvents and then the dilution scheme was performed in order to obtain the correct final concentration. A total of 50 µl of each concentration of the compound was added to columns 1 to 10, and then 50 µl of each concentration of MYR was added (ranging from 4 to 0.03 µg/ml) to rows A to G. Columns 11 and 12 were drug free controls. A fractional inhibitory concentration index (FICI) established the type of interaction which ranged from synergistic (FICI≤0.5), antagonism (FICI>4.0) and no interaction (FICI 0.5–4.0).

### Susceptibility of *G. mellonella* larvae to *Candida albicans* infection

Fifth instar *G. mellonella* larvae were used to investigate *Candida* pathogenesis and the effect of MYR during fungal infection. Twenty larvae were randomly chosen for each assay group, and all experiments were repeated three times. Each insect was injected with 20 µl of 10^5^ *Candida* cells, as described previously [24], incubated at 37°C and larval survival was recorded at, 24 h and 48 h post-injection. The larvae were considered dead when they showed no response to probing with a pipette tip. In addition, to show the potency of MYR as an immunosupressant during *Candida* infection, the insects were infected with *C. albicans* after pre-treatment with 10 times higher concentration of MYR (0.5 µg/ml) in comparison with the dose of MYR (0.05 µg/ml) used in the remained of the experiments. Larval survival curves were created using GraphPad Prism v5.0 (GraphPad Software, San Diego California USA).

### Effect of myriocin and antifungal agents on *G. mellonella* survival

*Galleria* larvae were pre-injected independently or in combination with 10 µl of 0.05 µg/ml MYR and 10 µl of the antifungal agents (AMPH or FLC) at a concentration of 250 µg/ml, then 1 h later injected with 10^5^ *C. albicans* cells. Survival rates were recorded at 24 h and 48 h post treatment. Control groups consisted of drug-free/PBS injected insects, non-injected, and drug only groups of larvae. Experiments were repeated three times (n = 20 each larval group).

### Recovery of *Candida albicans* from infected *G. mellonella* larvae

To investigate the effect of larval response and drug treatment on *Candida* growth recovery of fungal cells was determined. *Candida* was recovered from the fat body and hemolymph of insects (n = 10) 24 h and 48 h post injection with the above drug combinations or PBS (control). Hemolymph was collected in pre-chilled plastic tubes containing anticoagulant to which phenylthiourea (1 mg/ml) had been added to prevent melanisation. The anticoagulant comprised 62 mM NaCl, 100 mM glucose, 10 mM EDTA, 30 mM sodium citrate and 26 mM citric acid (pH 4.6) [25]. Fat body from infected larvae was homogenised in PBS and serially diluted. Aliquots (100 µl) of the fat body and hemolymph from each treatment were plated onto yeast extract potato dextrose (YEPD) agar plates containing streptomycin and ampicillin 1 mg/ml and incubated at 37°C for 48 h before recording the colony forming units (CFUs).

### Impact of MYR on the *G. mellonella* hemogram

Hemograms were determined to monitor the MYR effect on the cellular defences of *G. mellonella. Galleria* larvae were injected with *C. albicans* 1 h after treatment with MYR, AMPH or FLC drugs alone or in combination. Hemolymph was then recovered from 20 larvae, 24 h and 48 h post treatment, as described above. To determine the total hemocyte counts, 5 µl of hemolymph were mixed with 10 µl anticoagulant and phenylthiourea and numbers of cells counted using a hemocytometer.

### Effects of *G. mellonella* larval hemolymph and recombinant antimicrobial peptides (AMPs) on the *in vitro* growth of *C. albicans*

Larval hemolymph (n = 20), 24 h post treatment, was collected from different treatment groups (PBS injected, *Candida* alone, MYR, MYR-CA, AMPH-CA, FLC-CA, MYR-AMPH-CA, MYR-FLC-CA groups). A 10 µl aliquot of hemolymph (50 µl) and anticoagulant (10 µl) was spotted onto yeast minimal medium agar plates containing streptomycin and ampicillin (1 mg/ml) inoculated with 10^6^ or 10^5^ *Candida* cells. The cultures were incubated at 37°C for 48 h and inhibition zones (mm) recorded.

The antifungal properties of recombinant cecropin D and gallerimycin were determined using a turbidimetric assay [22]. The antimicrobial peptides cecropin D (Sequence: ENFFKEIERAGQRIRDAIISAAPAVETLAQAQKIIKGGD) and gallerimycin (Sequence: GVTITVKPPFPGCVFYECIANCRSRGYKNGGYCTINGCQCLR) from *G. mellonella* were produced by custom synthesis (Coring System Diagnostix, Gernsheim, Germany). Cysteine-free peptides were purified by reversed phase HPLC to >75% purity. Cysteine-containing peptides were used as raw products without further purification. Cecropin D and gallerimycin were dissolved in DMSO and used to determine antifungal activity. *Candida* cells suspended in yeast minimal medium were used at a final concentration of 2.5×10^4^ cells/ml. The recombinant AMPs at a final concentration of 500 µg/ml and fungal cells were incubated in 96-well microtitre plates (Greiner, UK) at 37°C and growth was determined spectrophotometrically at 600 nm. Readings were taken every 15 min over a 24 h period. Growth rate was calculated using the formula Y = N_0_+C*exp(-exp((2.7*μ/C)*(Lag-X)+1)) (N_0_ = initial number of cells; C = difference between initial and final cell numbers; Lag = time delayed before growth; *μ* = maximum specific growth rate).

### Impact of MYR on phenoloxidase (PO), Lysozyme, Superoxide dismutase (SOD) and malondialdehyde (MDA) activities

PO, lysozyme and oxidative stress management are important elements in insect immunity. PO, lysozyme, SOD and MDA activity were assayed as outlined below with each experiment being repeated three times unless otherwise indicated.

PO activity in the hemolymph was measured after 24 h post treatment in the following groups: insects injected with PBS, *Candida* alone, MYR, MYR-CA, AMPH-CA, FLC-CA, MYR-AMPH-CA, or MYR-FLC-CA. Cell-free hemolymph plasma samples from groups of 10 larvae were collected from treated and control insects. Hemolymph plasma fractions were prepared by collecting 10 µl of hemolymph from an incision made on the third proleg of each larva. This was diluted with 20 µl PBS and centrifuged at 500 g for 5 min at 4°C to remove the hemocyte pellet. The supernatants of the hemolymph plasma were then used for spectrophotometric analysis of PO enzymatic activity and protein concentration in a modification of the method described by Ashida [26]. Five microlitres of plasma were added to wells of a flat-bottomed 96-well microtitre plate containing 200 µl of 2 mg/ml L-DOPA (L-3,4-dihyroxyphenylalanine dissolved in sterile pyrogen-free water). After 30 min at 28°C, the absorbance was quantified at 490 nm using a plate reader (BMG Labtech, Germany). The units of PO activity were expressed as the change in absorbance at 490 nm per 1 min per mg of protein. The protein concentration of the samples was estimated by the Bradford method using BSA as the standard.

Lysozyme activity of hemolymph plasma from groups of 10 larvae was determined by a zone-of-clearance assay by using a substrate of freeze-dried *Micrococcus luteus* as a substrate suspended in agarose. The radius of the digested zone was compared with a standard curve made with egg white lysozyme (EWL) and expressed as an EWL equivalent (mg/ml).

Superoxide dismutase activity in the hemolymph from groups of 10 larvae was measured after 24 h post injection with PBS, *Candida* alone, MYR, MYR-CA, AMPH-CA, FLC-CA, MYR-AMPH-CA, or MYR-FLC-CA. Superoxide dismutase assay was performed as previously described [27].

Lipid peroxidation was determined using a modified–2-thiobarbituric acid (TBA) assay which quantifies the end product, malondialdehyde (MDA) [28]. Samples from groups of 10 larvae were tested.

### Implants inserted into *G. mellonella* larvae infected with *Candida albicans*

The encapsulation response is one of the frontline defences during pathogen invasion. Encapsulation responses were determined by implanting a 2 mm long, 0.5 mm diameter piece of white nylon monofilament through a perforation in the ventral segment of the cuticle of larvae infected with *C. albicans* and/or MYR, AMPH, or FLC. Implants were removed from the body cavity after 2 h, and then photographed from three angles. The degree of the melanization was quantified by image Pro software by measuring the coloration of all surface areas of each implant, and then comparing these values with that of a control implant prior to implantation (without melanization).

### Effect of MYR on the transcription of immunity and stress-related genes in *G. mellonella* infected with *Candida albicans*

Both AMPs and stress management genes play a major role in insect responses to pathogens [29]. The expression of 17 immunity and stress management genes was quantified in larvae treated with MYR, FLC, AMPH and *C. albicans* combinations, as outlined earlier. Fat body was recovered from at least 3 larvae per treatment at 24 and 48 h post-treatment. RNA was extracted from RNAlater stabilized fat body using a RNeasy Plus mini kit according to the manufacturer's instructions (Qiagen). RNA extracts were quantified spectrophometrically then reverse transcribed to cDNA using a qScript™ cDNA SuperMix (Quanta Bioscience). The cDNA quantity was checked and normalised using a reference gene PCR of 1/50 dilutions for each sample measured against a standard curve, and sufficient cDNA of similar concentration for each sample diluted to amplify all genes. Samples were quality checked for consistency between values for the two reference genes used: 18 s rRNA (AF286298) and elongation factor 1-alpha (EF1) (AF423811). Expression was then measured in the normalised samples using the Rotor-Gene 6000 (Corbett Research), with Rotor-Gene SYBR Green PCR mix (Qiagen), relative to these two reference genes. Cycling conditions were 95°C 5 min then 42 cycles of: 95°C 5 sec, annealing 10 sec, 72°C 20 sec. An initial touchdown of 1°C per cycle from 65°C for the first 5 cycles, resulted in optimal amplification for all loci. HRM analysis performed at the end of each run allowed each PCR to be checked for the presence of the expected product. All reactions were performed in triplicate, and optimal threshold values and reaction efficiencies calculated from 7-point serial dilutions of mixed cDNA from fungal- infected insects. Fold change values were calculated using the ΔΔCt method for each locus. The ΔΔCt for each sample was determined by subtracting the measured Ct value from the Ct value of each reference or ‘housekeeping’ gene. ΔΔCts were then converted to relative copy numbers with the formula 2^Δ^ΔΔ^Ct^. Fold changes were also calculated using reaction efficiencies using the *Pffafl* equation. Values showed similar trends for both reference genes and for each method of calculation: ΔΔCt values for 18 s are shown. Primers were designed from published *G. mellonella* sequences (NCBI) or from coding sequence where high homology protein sequences could be identified from an EST library [29], and are given in Table 1. For *hsp*90, a primer designed to the conserved 3′UTR region found in Lepidoptera [30] was paired with a degenerate primer designed from an alignment of 8 lepidopteran *hsp*90 sequences (GU230738, AB214972, AB060275, EF197936, GU230737, AF254880, GU230739, AB206477) using CODEHOP [31]. Details of the genes/contigs used are provided in the supplementary text (Text S1). Transcription data for all the treatments was presented in the form of heatmaps. Each cell in the heatmap shows mean gene expression fold changes relative to the uninjected larval control (basal expression). Specific color codes were used illustrate degrees of gene expression change relative to the PBS control, and relative to the *C. albicans* infected group at 24 h and 48 h.

**Table 1:** Gene loci and primers used to analyse gene expression in *Galleria mellonella* larvae.

Locus	Sequence reference	Putative Function/Process	Forward primer	Reverse primer
18 s	AF286298	Housekeeping	CACATCCAAGGAAGGCAG	AGTGTACTCATTCCGATTACGA
EF1: Elongation factor 1-Alpha (Ef-1a)	AF423811	Housekeeping	AACCTCCTTACAGTGAATCC	ATGTTATCTCCGTGCCAG
GAL: Gallerimycin	AF453824	Antimicrobial peptide	GAAGTCTACAGAATCACACGA	ATCGAAGACATTGACATCCA
GLIO: Galiomicin	AY528421	Antimicrobial peptide	GTGCGACGAATTACACCTC	TACTCGCACCAACAATTGAC
GLV: Gloverin-like protein	AF394588	Antimicrobial peptide	AGATGCACGGTCCTACAG	GATCGTAGGTGCCTTGTG
CER D: Cecropin D	Contig 19824	Antimicrobial peptide	CTGCGCCATGTTCTTCA	TCGCATCTCTGATCCTCTG
6tox	AF394584	Antimicrobial peptide	GCGAACTGCGAAGAATTATC	TGTCTGTCTTGAGTTGCATATTG
HSP 90	See methods	Molecular chaperone/stress response	GCRTCVCGYATGGAGGAAGT	GAACTAAATCAGTCTTTGG
TSF: Transferrin precursor	AY364430	Siderophore/antimicrobial peptide	CGTAGCAGTCATCAAGAAGG	CGCACTCACTAGAACTGG
18w: 18 wheeler	Contig14234	Toll Receptor	CACTCGATTTAGGCAACAACA	TCCGAGACGATCAACACTTC
IMPI: Inducible metalloproteinase inhibitor	AY330624	Metalloproteinase	TAGTAAGCAGTAGCATAGTCC	GCCATCTTCACAGTAGCA
C1-Contig 17373	Contig 17373	Glutathione peroxidase activity/Response to oxidative stress, Phospholipase A2 activity	CCACACTGTGAGGCAACATT	GTTTGCTTAGCACGGTCACA
C2- Contig 03093	Contig 03093	Peroxiredoxin activity/Response to oxidative stress	CTGACAATGACCGTGCACTT	TCTACGGGTGTAGCGACCTT
C3- Contig 15265	Contig 15265	G-protein coupled receptor activity/Stress response	CACACTGCAGGGCTTGTTTA	CCGTCCATCCTGACGTCTAT
C4- Contig 20595	Contig 20595	G-protein coupled receptor activity/Stress response	GCACATGACGTTAAGCCAGA	CCATTCCTGATCGCAACTTT
C5- Contig 21310	Contig 21310	Hsp protein binding/Stress response	GCAGCCTTAACGACCTGTTC	GTACACCTCAACCCCAGGAA
C6- Contig 1327	Contig 1327	Superoxide-generating NADPH/Oxidase activity/inflammatory response	GCTTGACATTGAGCTGTCCA	CCGTCCAATCACCTTTGACT
C7- Contig 15362	Contig 15362	Signal transducer/Regulation of cytokines, I-kappa B kinase/NF-kappa B cascade	CGAGCTAAAGACAGGCGATT	TCACCTGCGGTTGAATCATA
C8- Contig 19101	Contig 19101	Protein binding/Phagocytosis	ATTGCTAGCCAGGTTCAGGA	AGCTATTTGGCGGAAACTCA

Primers were designed from published *G. mellonella* sequences (NCBI) or from coding sequences where high homology protein sequences could be identified from an EST library.

### Statistical Analysis

All experiments were repeated three times with the data presented as the mean of those experiments. Statistical analysis of the data was performed using Prism v5.0 for Mac OS X (GraphPad software, San Diego California USA, www.graphpad.com). Data sets were tested for normal distribution using the Kolmogorov-Smirnov method. Deviation of outlier data was assessed by the Extreme studentized deviate method. Survival rates were compared using the Mantel-Cox test. Student's *t* test was used to analyze encapsulation response, and one-way ANOVA with Tukey's post tests were used for all other experiments. Significance levels were set at <0.05.

### Ethics Statement

No permits were required for the described study, which complied with all relevant regulations. The field studies did not involve endangered or protected species.

## Results

### MYR inhibits *C. albicans* growth *in vitro*

Interactions between MYR and the other antifungal agents were studied using a chequerboard microdilution assay. The minimum inhibitory concentration (MIC) for MYR in the presence of *Candida* cells at 48 h was 0.12 µg/ml compared with 2 µg/ml and 0.25 µg/ml for AMPH and FLC, respectively (Table 2). The MYR+AMPH combination was synergistic in inhibiting *Candida* growth at 0.25 µg/ml and 0.03 µg/ml, respectively, whereas the MYR combination with FLC was antagonistic with MICs of 16 µg/ml and 0.12 µg/ml, respectively (Table 2).

**Table 2:** *In vitro* activity of antifungal drug combinations against *Candida albicans*.

	Standard MIC (µg/ml)	Combinatio MIC (µg/ml)/MYR (µg/ml)	FICI	Interpretation
**MYR**	0.12	/		
**AMPH**	2	0.25/0.03	0.3	synergism
**FLC**	0.25	16/0.12	65	antagonism

Antifungal activity and interactions between drugs were determined using minimum inhibitory concentrations (MIC) and chequerboard microdilution assays, respectively. MIC was defined as the lowest concentration of antifungal agent resulting in an 80% inhibition of fungal growth compared with the control. Interactions based on FICI values were defined as: synergistic (FICI<0.5), antagonistic (FICI>4.0) and no interaction (FICI 0.5–4.0). MYR = myriocin; AMPH = amphotericin; FLC = fluconazole; MIC = minimum inhibitory concentration; FICI = fractional inhibitory concentration index.

### MYR reduces *G. mellonella* survival during *C. albicans* infection

**Figure 1:**
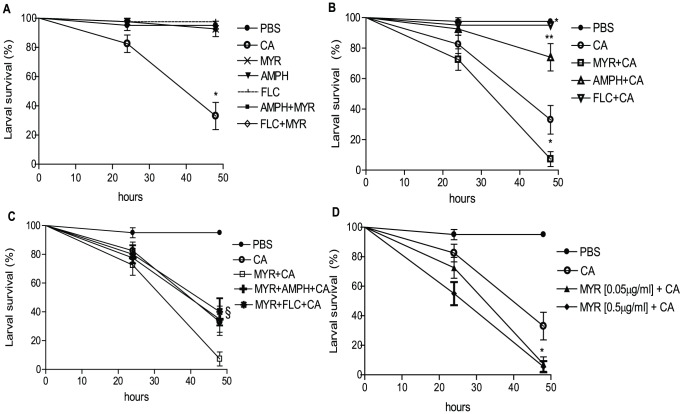
Effects of the antifungal drugs myriocin, amphotericin B, and fluconazole on *Galleria mellonella* survival 24 and 48 h post-treatment. (A) Comparison of the survival rates of *G. mellonella* larvae following injection with PBS, or antifungal drugs (MYR, FLC, or AMPH) alone or in combination, or with *C. albicans* alone. The survival rate of the *Candida-*infected group was significantly lower than the PBS control group (*p<0.0001, n = 20) and also compared with all other groups. (B) Comparison of the survival rates of *G. mellonella* larvae following injection with PBS, or *C. albicans* alone, or the antifungal drug followed by *C. albicans*. Pre-treatment of larvae with MYR, or AMPH or FLC had significant impacts on survival rates when compared with CA group (*p<0.001 and **p = 0.003, n = 20). (C) Comparison of the survival rates of *G. mellonella* larvae following injection with PBS, or *C. albicans* alone or with MYR combined with either AMPH or FLC followed by *C. albicans*. Larvae from combined groups (MYR+AMPH+CA and MYR+FLC+CA) showed increased survival rates (§ p = 0.03,and p = 0.01, n = 20), respectively, when compared with MYR+CA group. (D) Comparison of the survival rates of *G. mellonella* larvae following injection with PBS, or *C. albicans* alone or with two different concentrations of MYR followed by *C. albicans*. Effect of MYR at higher concentration (0.5 µg/ml) decreased the survival rate when compared with CA group (*p<0.001, n = 20). PBS = injected buffer; CA = *Candida albicans*; MYR = myriocin; AMPH = amphotericin; FLC = fluconazole.

Larvae of *G. mellonella* injected with 10^5^ *Candida* yeast cells resulted in survival rates of 82% and 33% at 24 h and 48 h, respectively, compared with 97% survival of larvae injected with PBS alone (p<0.0001; Figure 1A). MYR injection alone showed no apparent toxic effect that decreased larval survival below that of the PBS control at 48 h (Figure 1A). Larvae injected with the antifungal agents AMPH or FLC alone, also resulted in 97% larval survival at 48 h (Figure 1A). However, when MYR was injected into larvae which were subsequently infected with *C. albicans*, MYR treatment was lethal, reducing survival rates to 70% and 7% at 24 h and 48 h, respectively, and were significantly lower than *C. albicans* alone (p = 0.001; Figure 1B). In contrast, AMPH or FLC pre-treatments had a prophylactic effect, increasing larval survival rates, after fungal injection, for AMPH to 92% and 74% (p = 0.003) and for FLC to 100% at 24 h and 48 h, respectively (p<0.001; Figure 1B). In addition, the survival rates of insects infected with *C. albicans* after pre-treatment with MYR combined with either AMPH or FLC, were similar (p = 0.8) to the group infected with *Candida* alone (Figure 1C). However combined groups (MYR+AMPH+CA and MYR+FLC+CA) showed significant increased survival rates (p = 0.03 and p = 0.01; Figure 1C), respectively, when compared with MYR+CA group. Therefore, the anti-*C. albicans* synergy observed *in vitro* between MYR and AMPH (Table 2) was not observed in *G. mellonella* larvae infected with *C. albicans*. Increasing the MYR concentration ten-fold (to 0.5 µg/ml) did not improve antifungal prophylaxis in *C. albicans* infected larvae and instead mortality increased significantly at 24 h (p<0.001; Figure 1D) and at 0.05 µg/ml MYR concentration (p = 0.04) when compared with larvae from the CA group.

### *C. albicans* recovered from *G. mellonella* larvae treated with MYR

**Figure 2:**
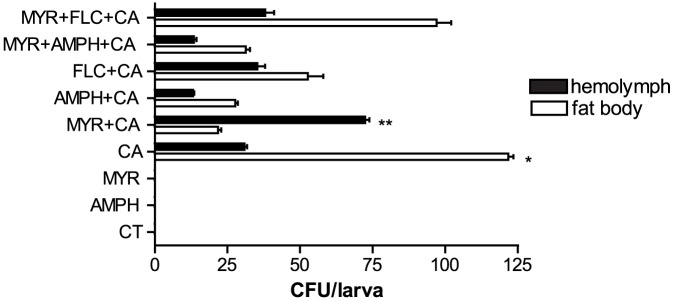
Recovery of *Candida albicans* from infected *Galleria mellonella* larvae. The number of *C. albicans* CFUs recovered from *G. mellonella* larval fat body and hemolymph 24 h post-drug treatment and infection. CFUs recovered from the fat body of the CA group were significantly higher than all the other groups (*p<0.01, n = 10). In contrast, CFUs recovered from the hemolymph of MYR+CA group were significantly higher (**p<0.001, n = 10) than all the other groups. In addition, only in the MYR+CA group were the CFUs recovered from hemolymph significantly higher than from the fat body (**p<0.001, n = 10). The drug treatments and abbreviations are as in Figure 1. Error bars indicate standard errors. Each experiment was triplicated.

To determine the cause of death of the *G. mellonella* larvae at 24 h, we examined the fat body and hemolymph of *C. albicans* infected insects treated with different combinations of antifungals (Figure 2). C*andida* cells were recovered from all infected insects with the highest numbers recorded from the fat body of the group injected solely with *C. albicans* followed by the MYR+FLC group (Figure 2). Total *Candida* CFUs recovered from the fat body and hemolymph were lowest in insects treated with either AMPH or MYR+AMPH combined (Figure 2). *Candida* CFUs recovered from the fat body of the CA group were significantly higher than all the other groups (p<0.01). In contrast, CFUs recovered from the hemolymph of MYR+CA group were significantly higher (p<0.001) than all the other groups. In addition, only in the MYR+CA group were the CFUs recovered from hemolymph significantly higher than from the fat body (p<0.001). At 48 h, the CFUs recovered from the fat body were significantly higher (p<0.001) than at 24 h in the MYR+*C. albicans* treatment (data not shown).

### MYR impacts on the *G. mellonella* hemogram

**Figure 3:**
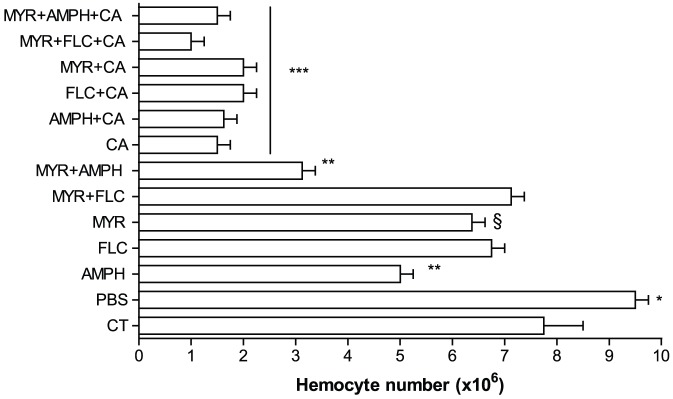
Impact of myriocin and antifungal drugs on the *Galleria mellonella* hemograms. Hemocyte numbers (cells/ml) of *G. mellonella* larvae were determined 24 h after injection with *C. albicans* and/or drugs. The PBS group showed a significant increase in hemocyte numbers (*p<0.001, n = 20) and all the other groups showed a decrease of the hemocytes in comparison with control (CT). In particular, AMPH and MYR+AMPH groups showed decreased numbers (**p<0.001, n = 20). In all the groups where *Candida* was present, the hemocyte numbers were lower (***p<0.0001, n = 20). Controls were uninjected (CT) or PBS injected larvae. The drug treatments and abbreviations are as in Figure 1. Error bars indicate standard errors. Each experiment was triplicated.

Injecting larvae with PBS resulted in a significant increase in hemocyte numbers compared with the non-injected control (p<0.001; Figure 3). When larvae were injected solely with MYR, FLC or AMPH, a significant decrease (p<0.001) in the hemocyte numbers was observed in comparison to the PBS injected controls (Figure 3). This reduction was particularly marked with AMPH and AMPH+MYR with counts significantly lower than all the other groups (p<0.001; Fig. 3). Following treatment solely with *C. albicans*, hemocyte numbers fell significantly (p<0.001; Figure 3) and remained low even in the presence of MYR, FLC, or AMPH used alone or in combination (MYR+FLC, MYR+AMPH; p<0.001; Figure 3).

### *G. mellonella* larval hemolymph and recombinant antimicrobial peptides (AMPs) do not affect growth of *C. albicans in vitro*

**Figure 4:**
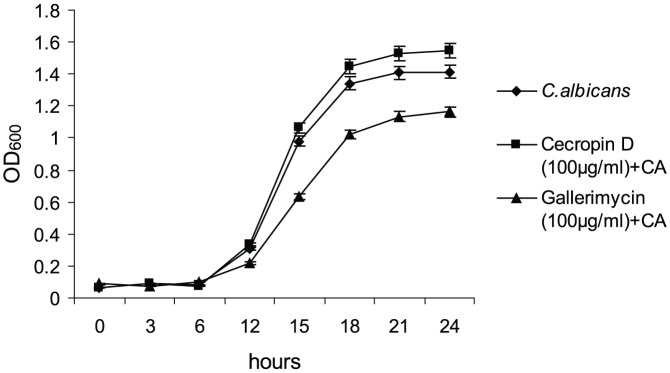
Effect of *Galleria mellonella* recombinant antimicrobial peptides on the *in vitro* growth of *Candida albicans*. The growth of *C. albicans in vitro* was monitored spectrometrically over 24 h to determine the inhibitory effect of recombinant antimicrobial peptides cecropin D and gallerimycin. The maximum growth rate for *C. albicans* was 0.11, for galleriomycin and cecropin D were 0.06 and 0.08, respectively. Error bars indicate standard errors. Each experiment was triplicated.

Hemolymph from larvae of *G. mellonella* pre-injected with PBS (control), antifungal drugs or *C. albicans* was tested after 24 h and was found to have no inhibitory effect on *C. albicans* growth *in vitro* (data not shown). The recombinant AMPs, cecropin D and gallerimycin were also tested *in vitro* against *C. albicans* and only the latter exhibited anti-*Candida* activity at 100 µg/ml (p<0.001; Figure 4).

### Impact of MYR on phenoloxidase (PO), lysozyme, encapsulation, superoxide dismutase (SOD) and malondialdehyde (MDA) activity

**Figure 5:**
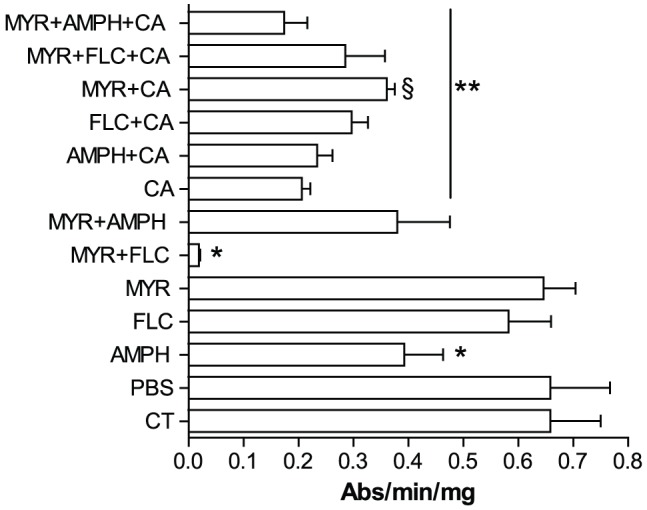
Impact of myriocin and antifungal drugs on phenoloxidase (PO) activity during *Candida albicans* pathogenesis. The *in vitro* PO activity of *G. mellonella* larval hemolymph was measured at 24 h during fungal pathogenesis and drug treatments. Significant decreases of PO activity were detected in the groups treated with AMPH and MYR+FLC (*p<0.001, n = 10) when compared with the controls. In addition, all the treatments in which *Candida* was present, a significant decrease of PO activity was observed when compared with the control groups (**p<0.001, n = 10). However larvae from MYR+CA group showed higher PO activity (§ p<0.05) when compared with CA group alone. Controls were uninjected (CT) or PBS injected larvae. The drug treatments and abbreviations are as in Figure 1. Error bars indicate standard errors. Each experiment was triplicated.

**Figure 6:**
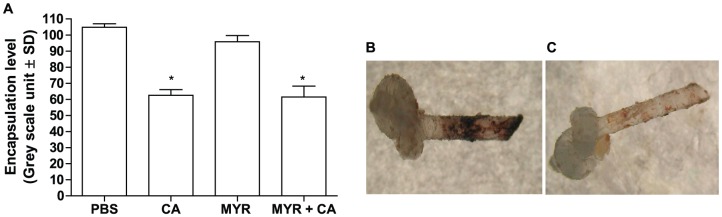
Encapsulation response of *G. mellonella* was decreased during *Candida* pathogenesis. Melanization of the implants was quantified to determine if encapsulation was affected during *Candida* pathogenesis after implants were introduced into the left pro-leg of larvae 24 h post- infection. Two hours after insertion of implants, during *C. albicans* pathogenesis (n = 5, triplicated), the degree of the melanization was quantified using Image Pro software by first measuring the coloration (gray value) of all areas of each implant, and then comparing these values with intact implants (not inserted). Decreased encapsulation responses were observed in larvae infected with *Candida* with or without pre treatment with MYR compared with PBS injected larvae (Figure A, *p<0.05). Plastic implants inserted into *C. albicans-*infected *Galleria* larvae showed markedly reduced melanization illustrated in the Figure C compared with PBS control insects (Figure B).

The effect of antifungal drugs on major immune components of *G. mellonella*, including PO, lysozyme and reactive oxygen species, were monitored. PO and lysozyme play a major role in insect humoral immune responses to invading organisms. Larvae injected with MYR or FLC alone showed no changes (p>0.05) in the PO activity in the hemolymph when compared with the control groups whereas AMPH alone significantly reduced PO activity (p<0.001; Figure 5). PO activity was also significantly lower (p<0.001) in all the treatments in which *C. albicans* was present as well as with the MYR+FLC combination in the absence of the pathogen (p<0.001; Figure 5). In addition, cellular host response was investigated by the encapsulation process during infection. Melanotic encapsulation of plastic implants inserted into *C. albicans*-infected *Galleria* larvae was quantified using Image Pro software by measuring the coloration of the area of the implants. Encapsulation was significantly reduced (p<0.05; Figure 6A), with and without MYR, AMPH and FLC, and showed only limited melanisation compared with untreated and PBS control insects (Figure 6B). All the treatments induced lysozyme activity. The highest activity was with *C. albicans* alone (p<0.001; Figure S1), then FLC with (p<0.001; Figure S1) and without fungus (p<0.0001; Figure S1), and then the MYR+FLC combination with *C. albicans* (p = 0.001; Figure S1). Lysozyme activity was higher in insects treated with *Candida* when compared with MYR or *C. albicans* plus MYR treatments (p<0.001; Figure S1). SOD activity was slightly depressed in the MYR+FLC group (p>0.05; Figure S2) than the PBS control, although this was not statistically significant. A marked increase in SOD activity was detected in the AMPH+CA and MYR+FLC+CA groups (p<0.01; Figure S2). The MDA content, except for MYR alone (p = 0.2), was higher in all treatments compared with the PBS and un-injected control groups, particularly in the presence of *C. albicans* (p = 0.001–0.03; Figure S3).

### MYR impacts on immunity and stress management genes during *C. albicans* pathogenesis

**Figure 7:**
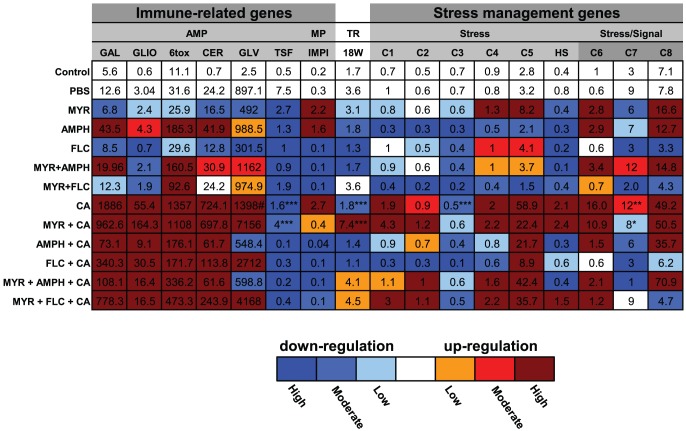
Gene expression comparison between PBS control and treated groups in *Galleria mellonella*. Expression of 17 gene transcripts in response to antifungal drugs and *C. albicans* 24 h post treatment. Mean value in each cell of the heat-map corresponds to gene expression fold change relative to uninjected larvae control (basal expression). Color gradient was used to visualize the gene expression comparison between treated groups and PBS injected control. The color gradients correspond to 3 different levels of gene expression relative to PBS injected control. A light color represents low gene expression i.e.0–25% of that expressed by the PBS injected control; intermediate color to moderate gene expression i.e. 25%–50% of PBS injected control; dark color to high gene expression, above 50% of PBS injected control. Among the 17 gene transcripts investigated during *Candida* pathogenesis (CA group) in the *Galleria* larvae only 3 genes TSF, 18W and C3 showed significant down-regulation (***p<0.001) in comparison with PBS injected control. The remaining gene transcripts in this group were significantly up-regulated (p<0.001). When the effect of MYR on the gene expression, during *Candida* pathogenesis (MYR+CA group), was compared with PBS control down-regulation profile on TSF (p<0.001) was found. Contrasting 18W and C7 transcripts results from CA and MYR+CA groups showed opposite gene-regulation profiles when compared to PBS control (***p<0.001, **p<0.05, *p>0.05). MYR = myriocin; AMPH = amphotericin; FLC = fluconazole, AMP = antimicrobial peptide; MP = metalloproteinase; TR = Toll receptor; GAL = gallerimycin; GLIO = galiomicin, 6tox; CER D = cecropin D; GLV = gloverin; TSF = transferrin; IMPI = Inducible metalloproteinase inhibitor; 18W = 18 wheeler; HS = heat shock protein 90; Contig codes for putative stress management genes Contig 17373 (C1); Contig 03093 (C2); Contig 15265 (C3); Contig 20595 (C4); Contig 21310 (C5); Contig 1327 (C6); Contig15362 (C7); Contig19101 (C8); #×10.

The effect of MYR treatment on a total of 17 genes was investigated. Differences in expression of AMPs (n = 6 genes), IMPI, 18W genes and putative stress management genes (n = 9 genes) were measured between drug-treated and PBS-injected groups (Figure 7). The colour gradient illustrated in the heat map (Figure 7) shows the corresponding percentage of down or up-regulation of the treatment groups in relation to the PBS controls.

In the MYR alone group, all AMPs (G*allerimycin, Galiomicin,* 6tox, *Cecropin D, Gloverin, Transferrin*) and 18W were down-regulated to different degrees when compared with the PBS injected group, while the IMPI gene showed up-regulation (p<0.001; Figure 7). However only *Transferrin* and IMPI showed statistical differences (p<0.001). Among the stress management genes Contig*s* 17373 (C1) (p>0.05), 15265 (C3) (p<0.01), 15362 (C7) (p<0.01) and hsp90 (p>0.05) were down-regulated while Contigs 20595 (C4) (p>0.05), 21310 (C5) (p<0.05), 1327 (C6) (p<0.01) and 19101 (C8) (p<0.01) were up-regulated in comparison with the PBS controls (Figure 7). Therefore, among the 17 genes investigated, 11 genes were down-regulated and 5 genes were up-regulated by MYR-treatment alone.

**Figure 8:**
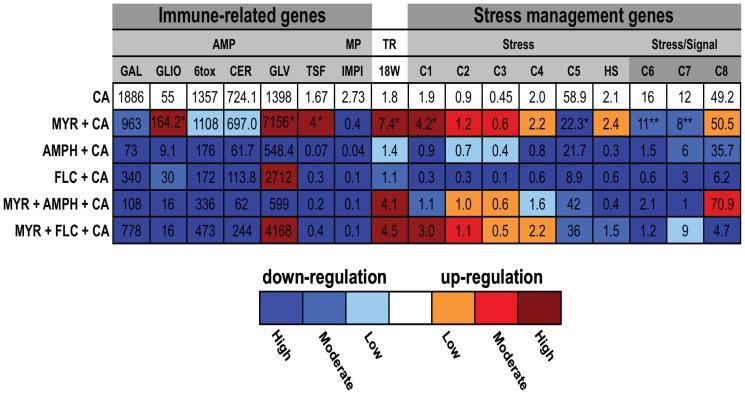
Gene expression comparison between *C. albicans* alone and drug-treated groups in *Galleria mellonella*. Gene expression comparing *C. albicans* infected larvae group with and without antifungals. Expression of 17 gene transcripts in response to *C. albicans* and antifungal drugs 24 h post treatment. The value in each heat-map cell corresponds to the mean gene expression fold change relative to the uninjected larvae control (basal expression). To visualize the change in the gene expression a color gradient was used. The color gradients correspond to 3 different levels of gene expression relative to CA group. A light color represents low gene expression i.e.0–25% of that expressed by *C. albicans* alone; intermediate color to moderate gene expression i.e. 25%–50% of *C. albicans* alone; dark color to high gene expression, above 50% of *C. albicans* alone. The effect of MYR during *Candida* pathogenesis (MYR+CA group) in the *Galleria* larvae in comparison with CA group, showed a significant increase (*p<0.001) in the level of gene expression of 3 antimicrobial peptides GLIO, GVL and TSF, toll receptor 18W and stress related gene C1. On the other hand this treatment also caused a significant decrease of gene expression of IMPI, C5 (*p<0.001), C6 and C7 (**p<0.05). Abbreviations listed in Figure 7.

When the antifungal drugs AMPH and FLC were tested on the insects different effects on gene expression were observed. AMPH treatment alone resulted in down-regulation of 9/17 genes and up-regulation of 8/17 genes compared with the PBS controls (Figure 7). Interestingly, virtually all (7/9) stress-management genes were down-regulated by AMPH treatment alone. FLC alone down-regulated 13/17 genes and up-regulated of only 2 genes among the 17 genes investigated (Figure 7). In general, MYR plus either AMPH or FLC caused changes in gene regulation to varying degrees when compared with drug treatments alone (Figure 7). With *Candida* infection alone, 14/17 genes (G*allerimycin*, *Galiomicin*, 6tox, *Cecropin D*, *Gloverin*, *IMPI*, *hsp90* and Contig*s* 17373 (C1), 03093 (C7), 20595 (C4), 21310 (C5), 1327 (C6), 15362 (C7), 19101 (C8)) were up-regulated with only *Transferrin*, 18W, and Contig 15265 (C3) down-regulated in comparison to the PBS controls (p<0.001; Figure 7). Pre-treatment with MYR followed by *Candida* infection also caused up-regulation of the same set of genes except that 18W and Contig 15362 (C7) which were up and down-regulated, respectively (Figure 7). Pre-treatment with MYR+AMPH or MYR+FLC combinations prior to *C. albicans* resulted in up-regulation of the majority of the AMP genes (Figure 7). These two groups also showed different levels of modulation of the stress management genes (Figure 7). Interesting results were obtained comparing fold changes of the genes between the *C. albicans* alone infected larvae with and without the antifungals. With the MYR+*Candida* group, 10/17 genes (p<0.001; *Galiomicin*, *Gloverin, Transferrin, 18W, hsp90*), (p<0.05 *Contig 17373* (C1); p>0.05 *Contig 15265* (C3); p<0.01 *Contigs 20595* (C4), *03093* (C7), *and 19101* (C8)) were up-regulated while the remaining genes were down-regulated compared with *C. albicans* alone group (Figure 8). The same 5 stress related genes were up-regulated in the two groups but had significantly higher expression in the MYR+*Candida* group (p = 0.001). In addition, the stress-signaling related genes Contigs 1327 (C6) and 15362 (C7) were down-regulated in the MYR+*Candida* compared with the *Candida* alone treatments (p<0.05; Figure 8). In contrast to the MYR+*C. albicans* treatment compared with the *C. albicans* alone, pre-treatment with AMPH or FLC or MYR+AMPH or MYR+FLC combinations prior to *C. albicans* resulted in down-regulation of the majority of the AMP genes whilst different levels of modulation were observed for the stress management genes (Figure 8). At 48 h, in general, for all treatments, there was a marked down-regulation, to different degrees, of all gene transcripts from the *Galleria* larvae (Figure S4 and S5).

## Discussion

In the present study, we show that neither MYR, FLC nor AMPH alone or in combination were harmful to the model insect, *G. mellonella*. AMPH and FLC are designed to target fungal not animal cells whereas MYR primarily targets mammalian cells and its role as an antifungal has not been fully characterized. MYR in combination with *C. albicans* resulted in a remarkable 93% larval mortality after 48 h. This is in contrast to AMPH and FLC with *C. albicans* which only caused 26% and 0% mortalities, respectively, after 48 h. Similarly, the combination of MYR with the other drugs enhanced larval mortalities. This did not correspond with the synergistic or antagonistic effects of these drug combinations in the *in vitro* test against *Candida*. A preliminary study with *Galleria* larvae injected with the entomopathogen fungus *Metarhizium anisopliae*, used in biological control [32], showed an increased mortality when larvae were pre-treated with MYR (unpublished data). This result is similar to the present study using *Candida* which suggests that MYR has a general immunosupressive effect on the host. Furthermore, both fungi were susceptible to MYR *in vitro* (unpublished data).

In addition, although there was no obvious antifungal prophylaxis observed *in vivo* using MYR with *C. albicans*, *a* preliminary study with the topical application (i.e. main infection route) of *M. anisopliae* on the *Galleria* larvae pre-treated with MYR was performed and an antifungal prophylactic effect was observed so that survival rates were increased (unpublished data). Therefore, there is the possibility that future derivatives of MYR could reduce the risk of mycoses in immune-compromised patients. There are many examples where drugs have dual functions leading to improved health care and reduced cost. For example, in the 1990s when a new HIV protease inhibitor was introduced for HIV combined therapy, this inhibitor also inhibited *Candida* pathogenicity related proteases leading to a significant decrease in candidiasis in HIV immunodeficient patients [12]. Another potential application of myriocin could be for topical antifungal treatment as in superficial candidiasis, in particular, in patients with chronic mucocutaneous candidiasis (CMC) who suffer from a selective immune deficiency with increased susceptibility to superficial candidal infections [33]. It would also be interesting to test the susceptibility to MYR of other aetiological agents of fungal disease in CMC patients, such as dermatophyte infections, i.e. *Trichophyton* and *Epidermophyton*, histoplasmosis [33].

In *G. mellonella* larvae treated with the different combinations of drugs and *C. albicans*, the humoral and cellular defenses failed to control the infection or protect the host from increased mortality. There was no obvious correlation between levels of activity of PO, SOD, MDA, lysozyme, AMPs or hemograms with larval survival when infected with *C. albicans*. For example, the total hemocyte counts were similar for combinations of *C. albicans* with MYR or FLC yet larval survival times were markedly different. However, an important finding was the impact of MYR on the *Candida* colonization profile, where an increased number of circulating *Candida* CFUs were observed in the hemolymph of the MYR+CA group than the CA group alone; these cells may rapidly deplete nutrients and release toxic compounds accelerating death. Furthermore, this finding combined with larval hemocytopenia could contribute to decreased larval survival.

Furthermore, no *in vitro* antifungal activity of hemolymph from larvae pre-infected with *C. albicans* was detected. The AMPs, even though their genes were highly upregulated, failed to inhibit *C. albicans*, probably due to the peptides being degraded by the fungal proteases. Recombinant gallerimycin, which is known to have antifungal activity, had limited inhibition of *C. albicans in vitro* even when used at relatively high concentrations.

The hemocytopenia, recorded in all treatments with *C. albicans*, probably results from either an abortive attempt to encapsulate the fungus or from cell lysis. Since the hemocytes are a source of PO this would also partially explain why there was a corresponding decline in the levels of plasma PO activity and a reduced encapsulation response. Dunphy *et al.* (2003) also noted that pathogenic *C. albicans* reduce nodule formation by damaging the hemocytes, allowing the fungus to colonise the host [34]. Recovery of *C. albicans* from *G. mellonella* larvae revealed that the number of CFUs from the hemolymph was significantly higher than from the fat body only with the MYR treatment. Since MYR has been shown *in vitro* to kill *C. albicans* then *in vivo* the drug could either have been inactivated in some way or removed from the hemolymph before it could make contact with the fungus. MYR is a structural analogue of sphingosine [4], a precursor of important signaling molecules and an essential component of eukaryotes cell membranes, which can readily bind to and increase the permeability of cell membranes [35]. Thus, in the present paper, injection of MYR prior to the *C. albicans* may have resulted in the drug binding to the inner surface of the hemocoel, and particularly to the extensive fat body, so that drug dilution in the hemolymph occurred and reduced the killing of the fungus to give the increase in CFUs and enhanced larval death recorded. In contrast, AMPH and FLC have different modes of antifungal activity to MYR as they target ergosterol, a key component of fungal cell membranes [36]. Following injection of AMPH and FLU into *G. mellonella* hemocoel, and in contrast to MYR, these drugs, because of their ergosterol specificity, would have remained at higher levels than MYR and available to kill *C. albicans*. This would then explain the higher survival rates of larvae with these drugs and the lower levels of *C. albicans* recorded in the hemolymph. Alternatively, exposure of *C. albicans* in the hemolymph to sub-MIC levels of MYR could have increased the lethality of the fungal infection by, for example, enhancing fungal protease secretion. However, pre-treating the *G. mellonella* larvae with a MYR concentration ten-fold higher (from 0.05 µg/ml to 0.5 µg/ml) did not improve antifungal prophylaxis in *C. albicans* infected larvae and instead mortality increased significantly at 24 h. This indicates that the fungal pathogenicity is not enhanced by sub-MIC levels of MYR.

Regarding the expression of the immune and stress management genes, all drug combinations tested affected to various degrees all 17 genes examined. The AMP genes were significantly up-regulated by AMPH alone, MYR plus AMPH and by all drug combinations in the presence of *C. albicans*. However, as stated earlier, this upregulation of the AMP genes had no obvious effect on the outcome of the infection. It is possible that the AMPs play a role in protecting the host from secondary infections by opportunistic microbial pathogens. Particularly noteworthy was the effect of the *C. albicans* alone and the MYR+*C. albicans* combination on the upregulation of the stress management genes. Although five stress management genes were up-regulated in the two groups, this expression was significantly higher in the MYR+*C. albicans* combination. This may be due to a number of factors such as MYR increasing the permeability of the insect cell membranes and/or interfering with cell signaling and inducing oxidative stress all of which are well documented for MYR in vertebrate systems [21]. The stress induced by MYR, together with enhanced survival of *C. albicans* circulating in the hemolymph in the presence of reduced levels of this drug, probably combine to increase the mortality of insects receiving the MYR-*C. albicans* treatment. In addition, stress management genes were also upregulated in *C. albicans* infected insects pre-treated with combinations of MYR with FLC or AMPH, which only marginally improved survival compared with *C. albicans* alone. These observations provide further evidence that MYR was the cause of the stress which was exacerbated by *C. albicans*. In marked contrast with the MYR combination with *C. albicans*, expression of all the stress management genes was downregulated in *C. albicans* infected insects pre-treated with FLC or AMPH suggesting fungal inhibition which correlates with increased larval survival. Finally, in the MYR alone and the MYR+*C. albicans* groups, Contig 15362 (C7) was downregulated and this gene has a putative role in signal transduction/cytokine regulation, I-kappaB kinase/NF-kappaB cascade and inflammatory responses [29].

The present study, using the *Galleria* model, demonstrates for the first time, an intricate interplay between immune and stress management genes in response to pathogens and therapeutics. Such interplay is well documented in humans [37], [38] so that the opportunity exists to identify common mechanisms involved in disease pathology in these disparate groups. The findings of the present study may have clinical implications in the use of MYR, or its analogues, to treat various sphingolipid-associated disorders. The dual immunosuppressant and antifungal properties of MYR failed to be translated into the *G. mellonella* model host during *Candida* infection. Furthermore, MYR stresses and impacts on the host and exacerbates *C. albicans* infection leading to enhanced host mortality. This increased host mortality highlights the potential clinical implication and health risks since *G. mellonella* has proven to be an effective surrogate for the murine model for evaluating microbial pathogenicity and drug therapeutics. However, in the present study, MYR was applied early in infection but candidiasis may cause hyperinflammatory responses at later infection stages, so that application of MYR or its analogues at these later stages may be beneficial and dampen hyperinflammatory responses. It would therefore also be very useful to study late stage infections in the *G. mellonella* model. Many correlations for *G. mellonella* and mice have been established for the pathogenicity of microbes, including *C. albicans*, *Aspergillus fumigatus*, *Pseudomonas aeruginosa*, and *Yersinia pseudotuberculosis* [19], [20], [39] as well as in the efficacy of licensed drugs [40]. It is known that *C. albicans* has its own mechanisms, including proteases, for suppressing the immune response of the *Galleria* larvae [41], and the upregulation by MYR of the stress genes is an interesting observation. Surprisingly, stress-related genes are not only involved in protecting cells from environmental stressors and pathogens [42] but can also be toxic under certain circumstances. For example, HSP90 which is upregulated by MYR+*C. albicans* if nitrated can induce cell death in other systems [43]. Thus MYR is not overtly suppressing *Galleria* immunity but covertly doing so by upregulating the stress genes, some of which participate in killing the insect.

Finally, this is one of the first times, as far as we are aware, that a therapeutic-pathogen combination has been found to be more harmful than the pathogen alone. Most therapeutics, including FLC and AMPH, targeting a range of bacteria and fungi in insect models, generally increase survival [44], [45]. Furthermore, the antifungal synergies observed with MYR *in vitro* were not translated in the *C. albicans* infected insect host. However, antifungal drugs have been shown to work synergistically with other therapeutics as reported between AMPH and 5-flucytosine against *Cryptococcus neoformans* in *G. mellonella* [16] and between FLC and an *hsp*90 inhibitor against *C. albicans* in *G. mellonella*. Most often, the synergies detected in the *G. mellonella* model can be reproduced in mammals [37]. This alternative model demonstrated to be an attractive tool to study therapeutic prophylaxis of antifungal drugs. The increased mortalities of the *G. mellonella* hosts following MYR plus *C. albicans* treatment are now the subject of further investigation.

## Supporting Information

Text S1 (DOC)

Figure S1 **Lysozyme activity.** The lysozyme activity in the hemolymph of *Galleria mellonella* larvae was measured 24 h after injection with antifungal drugs with and without *Candida albicans*. All the treatments induced lysozyme activity to different levels. AMPH, FLC and MYR levels compared with PBS injected group were higher (* p<0.01, n = 20). CA and FLC+CA groups compared with PBS injected groups showed markedly increased lysozyme activity (**p<0.001, n = 20). MYR+AMPH, MYR+FLC, AMPH+CA and MYR+CA groups did not show statistical differences when compared with PBS inject group (***p>0.05, n = 20). PBS = injected buffer; CA = *Candida*; MYR = myriocin; AMPH = amphotericin; FLC = fluconazole. Each experiment was triplicated.
(EPS)

Figure S2 **Superoxide dismutase (SOD) activity.** SOD activity (antioxidant defense response) in the hemolymph of *G. mellonella* larvae was measured 24 h after injection with antifungal drugs with and without *C. albicans*. MYR+FLC treatment resulted in a slightly decreased SOD activity (Mean±SD 0.46±0.026, *p>0.05, n = 20) than the PBS control, although this was not statistically significant. A marked increase in SOD activity was detected in the AMPH+CA and MYR+FLC+CA groups (**p<0.01, n = 20) when compared with PBS control. PBS = injected buffer; CA = *Candida*; MYR = myriocin; AMPH = amphotericin; FLC = fluconazole. Each experiment was done in triplicate.
(EPS)

Figure S3 **Malondialdehyde (MDA) activity.** The MDA content in the hemolymph of *G. mellonella* larvae was measured 24 h after injection with antifungal drugs followed by *C. albicans*. The FLC+CA and MYR+FLC+CA groups showed the greatest MDA activity (*p<0.05, n = 20) when compared with the PBS control. PBS = injected buffer; CA = *Candida*; MYR = myriocin; AMPH = amphotericin; FLC = fluconazole. Each experiment was done triplicate.
(EPS)

Figure S4 **Gene expression comparison between PBS control and treated groups in** ***Galleria mellonella*** **.** Expression of 13 gene transcripts in response to *C. albicans* and antifungal drugs 48 h post treatment. Mean value in each cell of the heatmap corresponds to gene expression fold change relative to uninjected larvae control (basal expression). Gene expression was visualized using a color gradient. The color gradients correspond to 3 different levels of gene expression relative to PBS control. Light color corresponds to low gene expression i.e.0–25% of that expressed by the PBS control; intermediate color to moderate gene expression i.e. 25%–50% of PBS control; dark color to high gene expression, above 50% of PBS control. MYR = myriocin; AMPH = amphotericin; FLC = fluconazole, AMP = antimicrobial peptide; MP = metalloproteinase; TR = Toll receptor; GAL = gallerimycin; GLIO = galiomicin, 6tox; CER D = cecropin D; GLV = gloverin; TSF = transferrin; IMPI = Inducible metalloproteinase inhibitor; 18W = 18 wheeler; HS = heat shock protein 90; Contig codes for putative stress management genes Contig 17373 (C1); Contig 03093 (C2); Contig 15265 (C3); Contig 20595 (C4); Contig19101 (C8).
(EPS)

Figure S5 **Gene expression comparison between** ***C. albicans*** **alone and drug-treated groups in** ***Galleria mellonella*** **.** Expression of 13 gene transcripts in response to antifungal drugs in *C. albicans* infected larvae 48 h post treatment. The value in each heatmap cell corresponds to the mean gene expression fold change relative to the uninjected larvae control (basal expression). To visualize the effect of drug-treated groups on gene expression during *Candida* infection a color gradient was used. The color gradients correspond to 3 different levels of gene expression relative to CA group. A light color represents low gene expression (0–25% of CA group baseline), an intermediate color moderate gene expression (25%–50% of CA group baseline) and a dark color represents high gene expression, (above 50% of CA group baseline). Abbreviations are as listed for Figure S4.
(EPS)
