# Supplementary material for: Myriocin Significantly Increases the Mortality of a Non-Mammalian Model Host during Candida Pathogenesis
Source: PLoS One. 2013 Nov 15;8(11):e78905. doi: 10.1371/journal.pone.0078905 (PMC3829820; doi:10.1371/journal.pone.0078905)
Supplement: Text S1 — (DOC) [file pone.0078905.s001.doc]

**Text S1**

The genome of *Galleria mellonella* has not been sequenced. The contigs were selected following the transcriptomic analysis of *G. mellonella*, as reported by Vogel *et al.* 2011 [29]. *G. mellonella* transcriptome was characterized on Roche 454-FLX platform combined with traditional Sanger sequencing. The complete set of sequences were subjected to a protein translated BLASTx search and a gene ontology (GO) analysis using Blast2GO. Contig sequences, the information about their open reading frames (ORFs), and sequence of the contig used for RT PCR amplification selected for the present study are presented.

**1. Contig21310**

TTTTTTTTTTTTTGTTATTCAATATTTATTGAGAAAATTGCGTTATCAGTAATGATAAACAATAAATTAATTAAATATTAGGATTTAAATAAGAAAAAAGACCATTTAAATCCTAAATACAACAAATAGCTTTACATTACAATGTTTTAATATTAAAAACGTAAATGTACAAACATAAACACTATAATATAGTAGAGTGTCAACACCAGTGTTATTTTTATTCACAAAGAGTTAACAAAAAGGAAGGCAGATGCATTTAGGCCCCGCGGTGCATCGGATATTAAATTGTGTATCTACATTAAACAATTCACTAACGCCAAGCTAAATTTATTGACTAGCAGCACCATCTACTTCCATTGTCTGCGTTTCTGCCTTATCACCTTTATCGTCCACTTGCATTACTTCTACCTTATTTATTTCTTTACCTTCTATTTGTTCCTTATTGCTATCTACATTTGCTTTCTTATCTTTACTTTTATCGCGTTTCTTCGTATTTTTAGCACCTTTAGTGGCCTTCTTTTCTTCTTTCTTTTCGGCCTCTTGTTTGGTTTCAGTTTTTTGTTTGGATGCTTCGTTATCTACAGGTCGCGTGTCAGTTACCTCAGGCTGGGCACCCTCTTTATTGGTTTCCAGTTTTTGTTCCTGTGATTTTGGCACCTCAGTTTTTTCATTAGCAGCCTTAACGACCTGTTCCTGAGCCTCTGGCTGAGCTGCCTGATGCTCATCAGTTTTAATTTCCTCTTTGGTATCTTTCATCTCTACTTCACCATTCTCTGTAATCTTATTTGTGTTTTGAACATTTTCAGCATTTTCCTTCATTTCAACATCATTTTGTGGTTGAGGAACTTCCTGGGGTTGAGGTGTACTTCCACCTTTGTTTTCAGCTTTAGCTTCTAATACTGCTATACATTTCTGAATACATTTAATGGCCTCCTTTCTTGCCTGCCTAATGTTTTCTTTACCGTCTGTTTCAATATTATCTAATTTAATGAGATTCCTCGTTAACATCTCATCTAGATAAATGTACTGCTTGTCTTTTTTAGTACCTGTGAAGTTTTCGACGTCAGTCATTAGGTTTAAGACATCTGTTTGAATACTAAGGATTTGAGAAATTGGATCATTAGCTGTTTGCTGTTGTGGTTTGGGCTGAG

***ORFs (NCBI ORF Finder)***

Frame From To Length

-3 328 1071 744

+1 718 897 180

-1 597 .. 725 129

+3 567 .. 674 108

***Sequence amplified by RT PCR***

Start Sequence

LEFT PRIMER 674 GCAGCCTTAACGACCTGTTC

RIGHT PRIMER 866 GTACACCTCAACCCCAGGAA

1. **Contig15362**

CGTCAATATGTCTGATACTTTTTAATTACTTCATTTAATTTATTTATTTAATATACCAGTTTACTTTGAGTACTTTACATATTTTATTTCAATAACTTGATTTTGTTTTCCTTTTTTCCTTAATCATAATTTAATATTGGAAGTATTTTCTATTTCCAATGGTATCATAAGTTATACAAATAGTAATTTAATTAGTTATATTACAGTTGATTATAAATATGTCCAATAATCAAGACGAAATTATTGAAAGGTCCCATGTAAAAAATTTACCGTATGCGGAATATCGAAATGCATTAAATTTTAATAAGGATCAATGTGAAATTATCGCTAAGTTGGCAAATATGAAAATATCATTCAATGAAACAAGTAAACCTGGAGAATTGTTAATGAAAATGTTATACAGAGGAGGATGTACTGTACAGAACTTTAGAAGATTAAAAGAAGCTGCAGAAAATGTTCAATCAATACTAAAATACTATATACCTCAGTCAAAAGTTGCCATACTCATAGGAAATGATAAATATGTAAATTTAGCAAAATTGGCAACACCATCCATAGATTGTGATTCTCTATCTTCAAACCTGAAGAACTTAGGATTTGTTGTTGTGACAATAAAAAACACAGCTAGCAGTGATTTAAGAGAAATATTATCTAAAATATTTAGCTACATCCCAGAGGATTCATATTGTTTCATTTTCTACGCTGGACATGGCTGTGAATTAGTAAATACAAAATGTATGTTGGGAATTGACTGCCCCACAGAAGATATAAAGTTAGAACACTGTGTTACTGAGAATTTTGTTTTAGAGCAAGTGTATAAGTGCAAACCGGATCTTTGTGTTTTAATTATGGATATGTGTAGGATAAACTTAGATAGGAATGCAAATCCACAGATCTATTCATCTATATGTACAGTAGAATCCTACAATGTACATAAAAATCTATTAATAGGATACTCTACTCAGTCATCAAAAGCTGCATATGAAGTGTTACAGATAGAATGCTCTACCACTATTAATGATACTTACGAGCTAAAGACAGGCGATTCAGCAAGGATTGTGCCCGGTGGTAGTCAATATGTAAATGCCTTATGCACAAGATTAGGAGACAATCTTGATATTAGTAGTTTGTTGGATGCAGTACATAGAGATGTTGAACATTCTATGAAGAAACAAAAACCAATAAAAGTTCAATGGGGTGTTGAGAAGAGATCATTGTATGATTCAACCGCAGGTGACGAAACTAGTGTTTTAAATGCATTAAAAGATTTTTCAAAACATTACAAGGATTATTGCCATGCTTTTTAAAATTTTATTTTT

***ORFs (NCBI ORF Finder)***

Frame From To Length

+3 219 1307 1089

***Sequence amplified by RT PCR***

Start Sequence

LEFT PRIMER 1028 CGAGCTAAAGACAGGCGATT

RIGHT PRIMER 1237 TCACCTGCGGTTGAATCATA

1. **Contig01327_1.f1.exp**

TAGTCACATGTTGCACATTCTCGTTTACGCGCGCTGGTCATGGCAGCTCAATACACGTTCGAGATTTTTATTACTATCGAGTGAAAAATAAAATAAAAAAATGCAGGAATTTTTTTAATTTTTGAATTTAGAAATTTAGGAAATGATTTTTCTTCGGGGGAAAAAATATAAATAGAACAAAAGATAAAGAATGAGATTAATACCACTTTTAAAAGATAGTTATTATTTTTTCAAGAGACGTTTTTTTTGATATTATGGATCTCATTAGTGACGTATATTTTCTACAATACATACATGTTTTATTTTAATGGACGCCAATTTTATTATATAAGAAGAATTCTAGGGTTAGGATTATGTATATCCCGTGGCACGGCATCAGTTATCAATTTATGTTGCGCTTTGGTATTACTTCCGCTGTGCAAGAAACTCAACCAAGTACTCTACCGGATCCTATCGAAATTGTGGCCGAATTTATTTTTCTTTTGGTTGGAGAGAGCGAAGAGTTTCCACATGACAGTCGCTGTAACTCTTGTGATGTTTGCTTTGGTTCACTCCGTTTCTCACTTTGTCAATTTGTGGAATTTCTCGAGAAGTTATGATGAGAGAATGAAGGAGATCAATTTTGCTAAATATAAAAATGAAAGTCCATTTTTCTTATTGCTAAGTCAGCCTGGATTGACTGGTGTATCGATGTTGATCATTATATTGTTGATGGGAATGACCTCTATGAGAGTAGTACGGAGAAATATTTACAACGCGTTCTGGTACACCCATCAGCTCTACATGCTGTTTATAGCTTTACTAATAGTTCATCCCCTAAGTGGTGTGTTAAAAGAAGAAATATTAGATGATGTCGAATCTAGTCCTATTACTAGTCATGAAGAAGGCTGGAGCAATAGCTCGTCAATAGATACTCATAAGTTTATTTCAATCAGATCAAAGACTTGGACATGGATGGCGTTTCCGCTTGGATGCTTTTTGATCGATCTACTTTGGAGGATATCGTCACGAAACCGTGCAAGAGTGCATATTCTAGAAGTAACTCACATGCCGGGACGGACTCTAAGCTTGACATTGAGCTGTCCACACAATCAGTTCATGTGTAGAATGGGACAGTACATTTTGCTTCAGTGTTTGGATATATCACTTTTAGAGTGGCATCCCTTTACTGTTGTGAAGGTACCCACATCTAATTCAAGAAATTTCGTTGTATGGGTCCGAGTCAAAGGTGATTGGACGGAACATTTAGAAATGCTATTATTGGAAAATGGCGCTAATAGATTAAGTTTTCTAGTGGATGGTGCGTTCTCAAGCCCGATGGAGGGCGCAGCTGCGGACGAAGTGGCGTTATGCGTGGCCGCCGGTGTTGGCATCACACCATTTGTATCACTGTTACATCACATGTTACTGAAGCCAAGAACCAAATTACCGGGCAGAATACATTTGTTATGGATTGTTCGAACTGAGGAAGAAATTACATGGCTCGCTGATTTAGCAAATGACACTATATTGCAACTGAGAGACGCTAACCGACCTGACAGATTACATATAGAATTTTATGTAACAGGCACAAAAGGAAATGATATCAAAGCATTTGAAGGTAAAGAAGAATGTTCTGCAACTCATATGGTAGTTATTAATGAAAAAGGCAAAATAACTCATGCATTATCTAATAATGAGAAAAGTATGACGGATGACGAAAAAGCAAGTCTTCTAACACCAAATAGAAGAAGACATGCTTGTACTGATGATGAAAAACGGAATAATTTAAACCATATGAAATACTATGAAATCGCAAAAGAATATCCTTTATTGGGATGCAGATTGAAAAGAGGTAGACCACATTGGGATCGCGTATTTGGATATTGGGTGCATTTATATCCCCACAAACGACTGAATTTATATTGTTGTGGGACAAAAAAATTAGTGAAATCATTGAAAAATAAATGTAAATATATTACTAGTAATACAAAAACCAAAATCACGATAGTTCATGAAAGATTTTCTTAATAAATAAATGTATTTGTATTCTTTTTATTACAATACAGTGTGTTGATTCAAAAAAAAA

***ORFs (NCBI ORF Finder)***

Frame From To Length

+1 295 2010 1716

-1 1670 1936 267

-3 1128 1373 246

+3 354 599 246

+3 951 1106 156

-1 932 1075 144

+3 3 125 123

-2 1543 1662 120

+3 1350 1466 117

+2 1298 1411 114

-2 661 774 114

***Sequence amplified by RT PCR***

Start Sequence

LEFT PRIMER 1067 GCTTGACATTGAGCTGTCCA

RIGHT PRIMER 1240 CCGTCCAATCACCTTTGACT

1. **Contig17373_1.exp**

TTTTTTTTTTTTTTTTTTTGCTGCAAAATGTTTTTAATTCGAAATATCTCTTAAGTACCTTTTATAAATTGACTATTACGATAAAATTTAAGAGAATACTATTAAACACACCATAAAAAAACTACATACTGTAAAAAAGATTTCTAGGTAAAATAAAAACATGATGATAAAATGCACTTTATAACATTAAAAATTAAAACTTGTATTTTTAATTTAACATTTTAGTTCTTTCATCAGTAAAATGTTACCTACACGCACTTTGAATGCTAAAGTAATAGGAGTTGATTATTATCAAGAATGTCTTCACCAATATTTTTCCAAAGACTTGACCAAATCTTTAGGATCTACATTGGGTCCATGTCGTTCAACAGGAACTCCGTCCTTGTCCACAATGAATTTCGTAAAATTCCACTTAATTGAACTGCCCAGGGTACCGCCTTGTTTATGCTTTAAGAATTTCCATAATTGGCTAGCATTGTCCCCATTAACATCGATTTTTTCAAACAAATCGAATTTCACTTTGCGATCAGCAGCGAAACAAACGATGTCTTCGGAGTTTCCTGGTTCTTGACCGGCGAATTGGTTGCAGGGAAATGCCAGGATTCGCAATCCTTTAGTCTCTGCATATTGTTCGTATAGTTCATTAAGTTGTTTGTAGTTATTTGCAGTAAGACCACACTGTGAGGCAACATTCACAATAATACAAACGTGACCTTTGTATATATCTAGTTTTACATCAGCACCTTTTATATTTTTGACGGTGAATTCATGAATAGACGTAGCCGACTTAAAATCGGGATTATCCACTGCCATTTTGCTGCAAAAAAGTATTGTGCTAAATTGTGACCGTGCTAAGCAAACTACATTACTAATAATAGGTACAATAAGCTTCGATACCGCTTTAGAACCAATTGTCATAGTGGACGGTATGTCTTATTTAAAGGAAAAATGAAAGTAACTATATCACTTATCACCATAAATCTATTGCGGTGATAAAATAGCAATTGGTTACACTTCGATTACACAACAATACAATACAGATTACTTAGAAAAAAA

***ORFs (NCBI ORF Finder)***

Frame From To Length

-1 304 918 615

+2 242 571 330

-2 516 692 177

+1 298 420 123

***Sequence amplified by RT PCR***

From Sequence

LEFT PRIMER 674 CCACACTGTGAGGCAACATT

RIGHT PRIMER 861 GTTTGCTTAGCACGGTCACA

1. **Contig20595_1.exp**

AAAAAAAGAATGTTTTGTTTTGTACCTGTTTCTACTATTTATAAAAGTACACAAATATTATAATTATAGTAAAAAATATTTGCTAAATAGTCAATACCGATAATAATTTCATGAAATACAAAGTAAATGTGCGTAAATTTTAACAATGATCTTAATTTTATGAAAAGTATCTTAAAATAACTACAGGCTAAAGATAATAATAAAAGCATTTGTTGTGAAACTGACTAGCAAATTTTGACAACATCGGTGCCGTACATTTATTGATGAGGATTCTTTTGTATGAATATGACGTGATACGTAATCTTTCATTCAGTGTAAATCTTTTAACCGCACGCCAGGGTCATCGGAGGACAGTCTGCTTTCTTCTGTGATAGCAGTGCTGGTCATGTTGCCACTCTTTCTGGAGTCCGTCGGTATGTGAGGTAGACCGCGGATGCTACGGTACCTTCTGACAATCAAGTTGCGCACTTTCTTCTTCCAAACAAAGATCATAAATATGAAGAAACCAAGCATTATGTTTGAGATATCGATTATCACCCAATACAAGTTGGAGCCACCGACGGCGAAACTGATCAGTTCCACAGACCAATTTATACCCATCACGACAAACAACTTCAGGTATAAGGAAAATCTCTGTTTGTCATTCTTCATTTTGTCTTTTGATGTTGACTTGGATCCCTTTAAAATTGAAGTCTCCTGTTTGATGGAGCGTATACGATGTGCTGTCACCGAGAAGAACACTATGTTGCATACAACCAGCACTAGGACTGGTGCGAAGAAGTATATCAGCTCACTCAGCCAATTGTCGAACCAACACCTCCGGTCTCCAAAGCCTGGCTTGATAACGCTGGGTGGCAAGTTAGCGTATTGCATGCCGATGGTAACTGCAGTCAATAGAAACGGAATACCCCATGCGTATGCACTGTATATGGTGAAACGTCTTGTTTCCCGGCGCTTTGATGTACTGCTGCCTCGGTAGCCACTGAACGTCCTCCAGATATCGAAGCACATGACGTTAAGCCAGAAAAAGCTCGACTGGAATGCGTAGTATACGATGAAAGCAATAATTACACATCCTGTCATAGCTATCCTCATTTGACCAGACTGAATCTTCAGGTATGCCAGGAATATGTAGGCCACAATAAGACCTGAGCAATACGCCATCAGGCACATACCATGTAGGTTTCGTAGCTCAGGAATGAACGCGTACACAACAAAAGTTGCGATCAGGAATGGCACGGATAATAGAAGT

***ORFs (NCBI ORF Finder)***

Frame From To Length

-2 310 1173 864

+2 434 979 546

-3 39 209 171

-2 181 309 129

-1 641 751 111

+3 264 371 108

***Sequence amplified by RT PCR***

**From Sequence**

LEFT PRIMER 1006 GCACATGACGTTAAGCCAGA

RIGHT PRIMER 1236 CCATTCCTGATCGCAACTTT

**6.** **Contig15265_1.f1.exp**

AGTTAGCATGAGAATTTGGGTGGTGTTACACGCATATTCTGCTAACGTTATTGTGGCCGTATTTATTTATGTGTCAAAAAGTTCGCGCACTATGAATACATAACAAATTACAGTTTTATTAATAAATAAAATAATACTTTGTTTCTGATATGATTATTTGTGGTATGAAGTGGTTTTGTGATGAATTTATTTTAGTGCAGTGAAAAGATTTTGTGCATTTATCGAATTTGGTGATTTGTCATCACATATATTTTTGTTTTCTTTTTACAAAAGAATTACAGAACAGCTGATACTCAATACTACAAAAAATATATCTTAAACCTAAATTAAAGAATACAGAAGTATTCCATTTAAAAAACCTAAGACACACGTCTGCTACAATAATTAAAATAATGAAAGAAAAAATAATAATAATTAGTCTGGTAGTGGGCTATTTCCTACCAAGTATGACCAGTGGGTATAACAACAATTGTTGCGACACTGGTCAACAACTAAAAAAATCATAAGTGAAGATGGATTTACTGTAAATATACTATGTTTGGACCTTGCAACAAATAATACCACACCATTATCTATAACATGCAAGACGCCGGTCGCAATACTGGCAACATTTTATATAAATGATGAAGGCCACTTAGTTGTGCAGACTTCGGTAAATCATGATATTATCATAAAGTCAGGAGAATATTGTTTGTACCAAACATTATATACTTTGAAAGTATACAACAGAGTCATATTATGTACAGATGACAACGAGGATAAGAATGACATTGCTGATTCAGTAAAAGGCTACTGTATGTTGGTATCAGTCATATTTCTTATATTGACTGCAACGGTATATGCTGCTATTCCTGAATTAAGAGACCTCCTCGGCAAAAGCTTGATCAGTTTCTGTGGAAGCTTATCTATCGGCCTGTCGATATTGGTCATCATGAAATTGATGGCATATTCGAATATGTCACTTTGCGCAGTGAGAGGTTTTTTCGCATATTTTTTTATACTGTCGAGTTTCTTCTGGTCTAATGCAATGGCGATACAAATAATGTTTAGTATGAGGCGACCATGCCTGTTATATGACCGTGGATGGAGAGAGTTCTCGTGGTACTCTTTATACGCGTGGGGGTGTCCGGCCGTGCTTACAATAATCACGGCGATTGTCAACTTCCACCCCGGCGATCACCCCAAGCCTGGCATTGGATTGATGCACTGCTGGTTTGTAGGAAATCAACAATGGTACTACATGTACAGCGTGATGACAATTTTAATATTGGCTAATATTGGGATCTTCATTTGGACTTCAACCCGTTTTTGGTGTTTATCCTTTAATTCATCCCATGTTAAAGCCGTGAAATATAAGCTCATGCTTACGATTCGTCTATTCGTACTGATGGGAATACCTTGGATATTTGAAATGATCGGCTCGTTGGTAGAAACAAGTATAGTATGGGCAATAATAGATATAATCAACACACTGCAGGGCTTGTTTATATTCGTATTGCTAGTGTTGTTGCGGCGGCGTGCCATAAAAATGATGCTAAAACACGGCTGGTTGAATTGTGTGTCGGATAGCATCGAAAAGTATTTAGCACTCGCTGAAGACGAAGAAGATGTTGTCGAGCATACGATAGACGTCAGGATGGACGGTAACATTACGAACGTAAGAAAGTATTGTTATAAAATAACAAAGCATTGATTTTATGTTAATGTAAAATGGTACAAACTAAGTAATTCGAAACGAGAAACTATCCGCGGATAGGTACTAACATTTTGACATCGTGAAGTTATGTTGAAAGGAACTTAATTCACAGTAATTAAGTATTGAGACTGTAATTTACAAAGAAAACGGACGTTTTGCAGAAATAATATACTCTAAAAAATGCGATTTCAATTTTGAATACATCGCAAGATAAAGAGATGTTACGTAAAATATTTACAATGAAGGTTACATTCTAAATATTGGATGTTTACGTATAGATTTAGGTTCGCAACAAATGTTTAGCTCCGTAAAGAAGAATGGCAGAATCTATCATAGTAAAAG

***ORFs (NCBI ORF Finder)***

Frame From To Length

+2 797 1693 897

-2 1237 1650 414

+1 1084 1275 192

-2 790 933 144

+1 1444 1569 126

-2 658 771 114

+3 393 506 114

-1 1583 1690 108

***Sequence amplified by RT PCR***

**From Sequence**

LEFT PRIMER 1468 CACACTGCAGGGCTTGTTTA

RIGHT PRIMER 1644 CCGTCCATCCTGACGTCTAT

**7. Contig19101_1.f1.exp**

CCTTTGTAAGTCTTTCAGCAGAAGGCGCAATACCATTTAATGCTGAAGAGTATTCTGATATAAATGAACTTCGAGCCTTAGCTGAATTGATTGGGCCATATGGCATGAAACTATTGAGTGAAACTTTGATGTGGCATATTGCTAGCCAGGTTCAGGAGCTGAAGAAGTTAGTTGTGCAGAATAAAGAAGTGCTCCAAATGCTACGAACTAACTTTGACAAACCTGAAATCATGAGAGAACAATTCAAAAGATTACAACATGTTGACAATGTATTGCAAAGAATGACAATAATCGGTGTTATTTTGAGTTTCCGCCAAATAGCTCAAGAGTCCTTATTAGATGTTTTAGAAAGACGAATTCCATTTTTGATCAGTTCAATTAAAGATTTCCAACAGCAGTTACCTAGTGGTGATCCTATGCGGGTTATATCAGAGATGTGTTCAGCAGCTGGTCTGGCCTGTAAAGTAGATCCAACATTAGCTACTTCATTGCGACAACATAAAGCGGAATCAGAGGAAGAGGAACACTTGATTGTTTGTCTACTAATGGTTTTTGTAGCAGTATCATTACCGAGATTGGCCCGCAGTGAAGGTTCTTTTTATAGGCCATCTTTGGAAGGTCATGCTAACAACATACATTGCATTGCGCCAGCTGTCAATCACATATTTGGTGCGCTATTCACAATATGCGGACAAGGCGACATTGAGGATCGTATGAAGGAATTTTTAGCATTGGCGTCTTCCTCTTTGTTGCGGCTGGGTCAAGAGACTGATAAAGAAGCAATAAAAAATCGAGAATCTGTTTATCTACTTCTAGATCTGATTGTTCAGGAATCACCATTCCTAACGATGGATTTATTAGAATCATGCTTTCCATATGTACTGATACGTAATGCTTATCATGAAGTTTATAAACAAGAGCAAATGTTATTACATTCATAATGAATTCATCTTTGTAAAATGTATTTTCTAAAAGACTTCACTTTGTATTAGAATAGATCACCTCCTAATTTTATTCATAAAAGCTATAGCTTAATTACGATTTATTTTTTGTCATCTAAGTAAAGATATCAATAACCAGGTATATTGCATTTTTTGTGAACATTGGGAGTTTATCTATGTAGTGATTAAGTATAGAAATCAAATTCCAATATTATTAAGACTAGTATTTTTAGCTTCCGTATTTTACTGAGATTATTTTATTTTGCTTCTCAAGCAATTACTATAAGTATGTAGAGTATGAATGAATTGGCATTGAAAACCACTCTTTTGCAAATATGATTAAGCTTTAACAAGACTGGTGAATTGTTTAAGTTATTAAATTGTGCATACATAAGCCCTATTATAAAACAGTACATTATATGTGTTAATAAAATTAATTTTAAAATAGGTATTGTTTTATCGCTTATTTTCTTATTTACTACTATAGTTCTCCCTATAGCCTATTCAAATTTTAATAATAAAGTACAGAATAATGTTTGCGTAATAAATTTATGTACACTGTTTGTCAATTGTCATTAATACGTAACGCAGGAAAATATTGCCTACATAAAATAGTTGTAAAATTCCCAATAACTTTTAGCTGTCACAAATATCTAATCACGCAAGAATTTTTATTTTTACATTACTTTGTTTTTTCACCGCAAGTATCTACTATCCGTAAATCAATTTACATCAGTGTCCTTCCTAGTCTCAGGATCTTTTGAGGGCTATATATAAACTATTAATTGGCACATTCCTCATTAATGATTTTATAGTTAAAATCACACAGTGCTGTATTAATTAAAATATAATTGCATTGAATTATGAATAGAAATTAAGTGGATTAAGGCACTAGATTAAGAACTATGTACATTATTTATTTAATTTTAACTCACAATGTAGAGGTACTGCCACATAAATATAATTTTATCTGAACTAAACATTTCTCTAATATTTTCGAAAAATGTTGTCAACCATTAGAGAACTGGTGAGTGCAATATACGGTGACGTCTGAATGAATGAATGCAATTTTATTGCACCGTCACTGTTACCTACATTGGTGTTAAAAAATTGATATCTTTTAGGGCTGGAAGAATACTGACAGCAGTCATGTTTCATATAATACAACAAATATTCACATTAGGGTATGTATTCACTAAGAGCTTTAACTCAAATTTACGTTACTATTATGTTTACGTCGATAATTTGTAGGTATCGAAAAGGTAACTGAGCCTGTAACGGGTGGTTTTTGAAATTCAGATTTACACATAAACCTATCGAAATAAACGTGAACGTAGACCTAACGTCTTTGTATAACTCACATTAGAAGCTCTGAATCGTTAAGACGGAAACGGTACATGCTTGCTTATATTGTATTTATTACGTAAAAATGCCTCTGGCTATTAGCATTATGTACCTATGTATTGCTGTACAAAAATTGCCTCACACTTCAGTAATAATATCGTATTTCAAGTTCGAGTTATTATTGATATACTCGAATGCTCGTGGTGAATATTGACCCTTATTTATGCGCTTCTTTCATTTTAACAAAGATTTTGTACCTTGTTTGTATTTACATAAGTATATTAATTATCTACCTGTTTAA

***ORFs (NCBI ORF Finder)***

Frame From To Length

+3 105 941 837

-1 679 939 261

+2 1493 1705 213

+3 2004 2192 189

-2 1929 2099 171

-3 1211 1357 147

+1 2371 2499 129

-1 481 609 129

+2 2480 2585 107

-3 1 106 106

-1 2146 2250 105

***Sequence amplified by RT PCR***

From Sequence

LEFT PRIMER 138 ATTGCTAGCCAGGTTCAGGA

RIGHT PRIMER 323 AGCTATTTGGCGGAAACTCA

**8. Contig03093_1.exp**

AAGCAGTGGTATCAACGCAGAGTACGCGGGAGTTTATGTATGTGTAAATACTTAATACCGCCGTCATCAAAAACATTTGAAATGATAATGTCTAATATTTAATTAATAAACCTCTTTTTAAGTAAAAAAAAATTTAAAACAAGAATTTGCAAATTTTTCTTCAAAAATAGGAATTGTGTTGAAGTGTCAGAACGATTTATTGCAAAAATTTTTATACCTGTAACCTAAAATGTTACTCCTGGGAGATGTTTTTCCTAATTTTACAGCAAGTACTACGGAGGGAGAAATAGTCTTTCATGATTGGTTAGGAGACTCATGGGGTATCCTGTTCTCCCATCCTTCTGATTTTACACCTGTATGCACAACAGAGCTTGCTCGAGTACTGAATTTATTGCCAGAGTTCAAAAAGCGCAATGTAAAAGTTATTGGCTTGTCATGTGACTCTATATCTTCTCATACAGAATGGTGCAAGGATATCAAATGTTATGGAGGTTACAATGAAGAATATAAGTTTCCATATCCAATTATTGCTGACAATGACCGTGCACTTGCAAGAAAGTTAGGGATGATGGATATGGATGAGTTGGATCCAGCTGGAATACCACTAACGGCTCGTGCTGTTTTCATTGTGGATCCCAACAAGAAATTTCGCCTGTCTTTTTTATATCCTGCTACTACTGGAAGAAACTTTGATGAGATATTGCGTGTACTGGATTCTTTGCAACTGACTGATAAAGCCAAGGTCGCTACACCCGTAGATTGGAAGATGGGAGATGACTGTATGGTGCTGCCGACTTTACCTGAAGACCAGGTGACCAAAGTTTTCCCACAAGGGGTCACAGTGGTACCCCTTCCCTCGGGCAAGAATTACCTGAAGAAGACTTCCTGCCCAAAGATATAAAATTTTCATATGATGATACTATGATATGATTTTGATATAATAATACTGTTATAAATTGTACCGCATGTACCTTATTTCATTGTATTTTTTTTAAAATAAACAAGTTAATTTTAAAAAAAAAAAAAAAAAAAAAAAAAAAA

***ORFs (NCBI ORF Finder)***

Frame From To Length

+2 230 901 672

-1 493 627 135

-3 797 910 114

***Sequence amplified by RT PCR***

From Sequence

LEFT PRIMER 531 CTGACAATGACCGTGCACTT

RIGHT PRIMER 759 TCTACGGGTGTAGCGACCTT
